# Cancer Incidence Among Adults With HIV in a Population-Based Cohort in Korea

**DOI:** 10.1001/jamanetworkopen.2022.24897

**Published:** 2022-08-02

**Authors:** Boyoung Park, Kyoung Hwan Ahn, Yunsu Choi, Jung Ho Kim, Hye Seong, Youn Jeong Kim, Jun Young Choi, Joon Young Song, Eunjung Lee, Yoon Hee Jun, Young Kyung Yoon, Won Suk Choi, Myungsun Lee, Jaehyun Seong, Shin-Woo Kim

**Affiliations:** 1 Department of Preventive Medicine, Hanyang University College of Medicine, Seoul, Republic of Korea; 2 Department of Internal Medicine and AIDS Research Institute, Yonsei University College of Medicine, Seoul, Republic of Korea; 3 Division of Infectious Diseases, Department of Internal Medicine, Korea University College of Medicine, Seoul, Republic of Korea; 4 Division of Infectious Diseases, Department of Internal Medicine, Soonchunhyang University Hospital, Seoul, Republic of Korea; 5 Division of Infectious Disease, Department of Internal Medicine, Seoul St Mary's Hospital, College of Medicine, The Catholic University of Korea, Seoul, Republic of Korea; 6 Division of Clinical Research, Center for Emerging Virus Research, National Institute of Infectious Diseases, Korea National Institute of Health, Cheongju-si, Chungcheongbuk-do, Republic of Korea; 7 Department of Internal Medicine, School of Medicine, Kyungpook National University, Daegu, Republic of Korea

## Abstract

**Importance:**

In combination with a decreased risk of AIDS-defining cancers and improved survival of people infected with HIV, the burden of non–AIDS-defining cancer has increased markedly. Although a substantial number of studies have measured the cancer risk among people with HIV in developed countries, little research has been conducted on the risk of cancer in HIV-infected people in Asia.

**Objective:**

To examine the cancer incidence and the estimated risk of cancer among people in Korea infected with HIV compared with the general population.

**Design, Setting, and Participants:**

This retrospective cohort study evaluated patients without cancer newly diagnosed with HIV from January 1, 2006, to December 31, 2018, using a nationwide population-based claims database embedded in the National Health Insurance Service database. Data were analyzed between December 6, 2021, and February 28, 2022.

**Exposures:**

Infection with HIV.

**Main Outcomes and Measures:**

Cancer incidence and standardized incidence rate (SIR) through indirect standardization.

**Results:**

A total of 11 552 individuals without cancer (10 444 male [90.4%]; mean [SD] age, 39.9 [11.2] years) diagnosed with HIV were identified. The SIR for all cancers was 1.68 (95% CI, 1.50-1.87) in men and 1.26 (95% CI, 0.89-1.64) in women. In men, the highest SIRs were for Kaposi sarcoma (SIR, 349.10; 95% CI, 196.10-502.20) and anal cancer (SIR, 104.20; 95% CI, 55.56-149.90). The incidence of non-Hodgkin lymphoma (SIR, 15.62; 95% CI, 11.85-19.39), Hodgkin lymphoma (SIR, 16.67; 95% CI, 4.32-29.02), and oropharyngeal cancer (SIR, 2.97; 95% CI, 1.36-4.58) in men infected with HIV was higher than in the general population. In women infected with HIV, an increased incidence of cervical cancer (SIR, 4.98; 95% CI, 1.29-8.66) and non-Hodgkin lymphoma (SIR, 11.78; 95% CI, 2.35-21.21) compared with the general population was observed. The SIR of thyroid cancer in patients with HIV was lower than in the general population in both men (SIR, 0.63; 95% CI, 0.27-0.99) and women (SIR, 0.48; 95% CI, 0.06-0.90).

**Conclusions and Relevance:**

In this cohort study, cancer risks, especially AIDS-defining cancer and virus-related cancer, were elevated in people with HIV. Efforts for cancer prevention, screening, and better accessibility to medical care in HIV-infected people are warranted.

## Key Points

**Question:**

What is the incidence of cancer in people with HIV infection in Korea, and does it differ from that in the general population?

**Findings:**

In a nationwide cohort study of 11 552 HIV-infected individuals, compared with noninfected individuals, the risk of any cancer, Kaposi sarcoma, anal cancer, non-Hodgkin lymphoma, Hodgkin lymphoma, and oropharyngeal cancer was higher in men infected with HIV, and the risk of cancer, cervical cancer, and non-Hodgkin lymphoma was higher in women infected with HIV. The risk of thyroid cancer was lower in all patients infected with HIV.

**Meaning:**

In this study, cancer risks, especially for AIDS-defining cancer and virus-related cancer, were elevated in people with HIV; the lower risk of thyroid cancer might reflect limited medical access for prevention.

## Teaser

This cohort study assesses the incidence and risk of cancer among people infected with HIV compared with the general population in Korea.

## Introduction

People infected with HIV are at an elevated risk of developing cancer. Three types of malignant cancers (non-Hodgkin lymphoma, Kaposi sarcoma, and cervical cancer) that are associated with the substantial progression of advanced-stage immunodeficiency (AIDS) are considered to be AIDS-defining cancers.^1^ The risk of other types of cancers, including lung cancer, anal cancer, Hodgkin lymphoma, oral cavity and pharyngeal cancer, hepatocellular carcinoma, and genital cancers, is also increased in people with HIV infection.^1^ Before the development of antiretroviral therapy, AIDS-defining cancers developed in more than 30% of people with HIV. With the introduction of antiretroviral therapy in 1996, in combination with a decreased risk of AIDS-defining cancers and improved survival of people infected with HIV, the burden of non–AIDS-defining cancer has increased markedly. Thus, in 2030, prostate and lung cancer, which are the most common cancers in the general population in the US, are projected to be the most common cancers in people with HIV infection.^2^

Although the number of new HIV cases has decreased worldwide, the number of HIV-infected cases has increased continuously in Korea since 1985, when the first case was identified. The distinct characteristics of HIV infection in Korea include an increased number of newly diagnosed cases, especially in the young age group, and a higher proportion of men diagnosed with HIV, accounting for approximately 90% of total HIV infections.^3,4^ In 2019, a total of 1222 new cases, including cases in 1111 men (90.9%) and 779 individuals 20 to 39 years of age (63.7%), were diagnosed and reported; the number of people living with HIV infection was 13 857.^3^ To reduce HIV transmission, full national health insurance coverage related to HIV treatment, a regular HIV screening test in the high-risk group, and a free anonymous screening system have been introduced in Korea.^5^ Because of the combination of high insurance coverage for HIV screening and treatment, including antiretroviral therapy, and younger age at diagnosis of HIV, long-term survival of individuals with HIV is expected. Hence, chronic diseases, including cancer, have emerged as health problems in HIV-infected people in Korea.^5^ However, although a substantial number of studies have measured the cancer risk among people with HIV in developed countries, there has been little research on the risk of cancer in HIV-infected people in Asia. Therefore, this study examined the cancer incidence and estimated risk of cancer among HIV-infected people compared with the general population using a nationwide, population-based claims database in Korea.

## Methods

### Study Design and Study Population

This retrospective cohort study was based on the health insurance claims data provided by the National Health Insurance Service–National Health Information Database (NHIS-NHID) in Korea. The NHIS is a mandatory health insurance system that covers all citizens in Korea, and information on the demographic characteristics, health care use, and deaths are included in the NHIS-NHID database.^6^ The entire medical history of people infected with HIV is available through analysis of the claims data. Both HIV infection and cancer are covered by catastrophic illness codes that are related to more cost-sharing of out-of-pocket expenses for rare diseases or diseases with a high financial burden. Thus, the combination of disease codes and catastrophic illness codes has high validity in defining conditions.^7^

All HIV cases were identified by HIV disease codes and catastrophic illness codes for HIV infection from January 1, 2006, to December 31, 2018. To identify newly diagnosed HIV cases, those who had any hospital visits for HIV with catastrophic illness codes before 2006 were excluded, leaving newly infected cases between 2006 and 2018. Furthermore, those with a cancer catastrophic illness code before the first date of visit attributable to HIV were excluded, leaving a remaining 13 238 people infected with HIV. In addition, those with more than 60 days between the first hospital visit for HIV and the date of registration for catastrophic illness codes, cancer diagnosis, or death within 90 days of first hospital visit for HIV were excluded to avoid possible prevalent cases. The study protocol was approved by the institutional review board of Hanyang University. Permission to use the NHIS-NHID database was approved by the National Health Insurance Sharing Service system, and anonymized data were available to the researchers. Thus, informed consent from the participants was waived. This study followed the Strengthening the Reporting of Observational Studies in Epidemiology (STROBE) reporting guideline.

### Cancer Incidence

Incident cancer cases were identified by a combination of cancer codes (C00-C99) of *International Statistical Classification of Diseases and Related Health Problems, Tenth Revision* (*ICD-10*) and catastrophic illness codes from health care use until December 31, 2019. The types of cancer were classified as AIDS-defining cancer and non–AIDS-defining cancer. AIDS-defining cancers include Kaposi sarcoma (C46), cervical cancer (C53), and non-Hodgkin lymphoma (C82-C86, C96). Non–AIDS-defining cancers include brain and central nervous system (C70-C72), urinary tract (C64-C68), breast (C50), colorectal (C18-C20), esophageal (C15), Hodgkin lymphoma (C81), liver, bile duct, and pancreatic (C22-C25), lung and tracheal (C33, C34), multiple myeloma and malignant immunoproliferative diseases (C88, C90), lip, oral cavity, and pharyngeal (C00-C14), stomach (C16), thyroid (C73), prostate (C61), anal (C21), and other cancers. Only cancer diagnosed 90 days after the first hospital visit for HIV was considered an incident cancer.

### Statistical Analysis

The incidence of cancer in individuals aged 20 to 79 years diagnosed with HIV was presented as the incidence rate per 100 00 person-years. The follow-up person-years at risk were estimated from the first hospital visit date for HIV to the date of cancer diagnosis, date of death, or December 31, 2019, whichever came first. The incidence was compared with that of the Korean general population from the Korea Central Cancer Registry through indirect standardization. Considering the changes in cancer incidence in the general population, we calculated the pooled incidence rate between 2006 and 2018. The standardized incidence rate (SIR) for all cancers and site-specific cancers was calculated as follows:

Standardized incidence rate = Observed cancer incidence in patients with HIV / Expected cancer incidence in patients with HIV.

The expected number of total incident cancer and site-specific cancer cases was calculated by multiplying the age-specific cancer incidence rates with 5-year intervals in the pooled general population for 2006 to 2018 and the number of person-years of patients diagnosed with HIV in each age group. The 95% CIs for the SIRs were calculated based on the Poisson distribution. A 2-sided *P* < .05 was considered to be statistically significant. All analyses were performed separately for men and women. SAS software, version 9.4 (SAS Institute Inc) was used for all statistical analyses. Data analysis was performed between December 6, 2021, and February 28, 2022.

## Results

A total of 11 552 individuals without cancer (10 444 men [90.4%] and 1108 women [9.6%]; mean [SD] age, 39.9 [11.2] years) were identified. Approximately 56% of HIV-infected men and 45% of HIV-infected women were aged 20 to 39 years old. The total follow-up years were 66 766.10 in men and 8090.87 in women, with a mean (SD) follow-up period of 6.4 (1.5) in men and 7.3 (1.3) years in women. The demographic characteristics of the patients diagnosed with HIV are presented in Table 1.

**Table 1.** Baseline Characteristics of Patients With HIV Infection 20 to 79 Years of Age in Korea, 2006-2018^a^

Characteristic	Men (n = 10 444)	Women (n = 1108)
Age at HIV diagnosis, y		
20-24	1370 (13.1)	78 (7.0)
25-29	1706 (16.3)	144 (13.0)
30-34	1432 (13.7)	134 (12.1)
35-39	1338 (12.8)	136 (12.3)
40-44	1306 (12.5)	99 (8.9)
45-49	1063 (10.2)	95 (8.6)
50-54	879 (8.4)	121 (10.9)
55-59	598 (5.7)	117 (10.6)
60-64	353 (3.4)	70 (6.3)
65-69	212 (2.0)	55 (5.0)
70-74	130 (1.2)	36 (3.2)
75-79	57 (0.5)	23 (2.1)
Year of diagnosis		
2005-2009	2551 (24.4)	388 (35.0)
2010-2014	3976 (38.1)	408 (36.8)
2015-2018	3917 (37.5)	312 (28.2)
Income level		
First quartile	1901 (18.2)	225 (20.3)
Second quartile	2749 (26.3)	266 (24.0)
Third quartile	2518 (24.1)	285 (25.7)
Fourth quartile	2755 (26.4)	231 (20.8)
Missing	521 (5.0)	101 (9.1)
Total follow-up years (mean)	66 766.10 (6.4)	8090.87 (7.3)

^a^ Data are presented as number (percentage) of patients unless otherwise indicated.

Among those diagnosed with HIV, 361 individuals developed cancer (317 cases in men and 44 cases in women) (Table 2). The crude cancer incidence rates were 474.79 (95% CI, 422.5-501.5) per 100 000 person-years in men and 543.82 (95% CI, 383.13-625.81) per 100 000 person-years in women. In men, the 5 most common incident cancers were non-Hodgkin lymphoma (100.35 [95% CI, 76.32-112.61] per 100 000 person-years), followed by liver, bile duct, and pancreatic cancer (49.43 [95% CI, 32.56-58.03] per 100 000 person-years), stomach cancer (44.93 [95% CI, 28.85-53.14] per 100 000 person-years), colorectal cancer (41.94 [95% CI, 26.40-49.86] per 100 000 person-years), and lung and tracheal cancer (41.94 [95% CI, 26.40-49.86] per 100 000 person-years). In women, cervical cancer was the most common incident cancer, with an incidence rate of 86.52 (95% CI, 22.42-119.22) per 100 000 person-years. Non-Hodgkin lymphoma (74.16 [95% CI, 14.82-104.43] per 100 000 person-years), liver, bile duct, and pancreatic cancer (74.16 per 100 000 person-years), and thyroid cancer (61.80 [95% CI, 7.63-89.43] per 100 000 person-years) were commonly incident.

**Table 2.** Incident Cancer Cases and Incidence Rate of Cancer in Patients With HIV Infection 20 to 79 Years of Age in Korea, 2006-2018

Cancer type	Men		Women	
	No.	Incidence rate^a^ (95% CI)	No.	Incidence rate^a^ (95% CI)
All cancers	317	474.79 (422.5-501.5)	44	543.82 (383.13-625.81)
AIDS-defining cancer				
Kaposi sarcoma	20	29.960 (16.83-36.65)	0	0
Cervical cancer	0	0	7	86.52 (22.42-119.22)
Non-Hodgkin lymphoma	67	100.35 (76.32-112.61)	6	74.16 (14.82-104.43)
Non–AIDS-defining cancer				
Brain and CNS cancer	2	3.00 (0.00-5.11)	1	12.36 (0.00-24.72)
Urinary tract cancer	9	13.48 (4.67-17.97)	0	0
Breast cancer	1	1.50 (0.00-3.00)	4	49.44 (0.99-74.16)
Colorectal cancer	28	41.94 (26.40-49.86)	2	24.72 (0.00-42.20)
Esophagus	3	4.49 (0.00-7.09)	0	0
Hodgkin lymphoma	7	10.48 (2.72-14.45)	1	12.36 (0.00-24.72)
Liver, bile duct, and pancreas cancer	33	49.43 (32.56-58.03)	6	74.16 (14.82-104.43)
Lung and tracheal cancer	28	41.94 (26.40-49.86)	3	37.08 (0.00-58.49)
Malignant immunoproliferative diseases	5	7.49 (0.92-10.84)	0	0
Oropharyngeal cancer	13	19.47 (8.89-24.87)	0	0
Stomach cancer	30	44.93 (28.85-53.14)	4	49.44 (0.99-74.16)
Thyroid cancer	12	17.97 (7.80-23.16)	5	61.80 (7.63-89.43)
Prostate cancer	17	25.46 (13.36-31.64)	0	0
Anal cancer	20	29.96 (16.83-36.65)	0	0
Others	26	38.94 (23.97-46.58)	5	61.80 (7.63-89.43)

Abbreviation: CNS, central nervous system.

^a^ Per 100 000 person-years.

The SIRs of total cancer were 1.68 (95% CI, 1.50-1.87) in men and 1.26 (95% CI, 0.89-1.64) in women, indicating a significantly higher cancer incidence in HIV-infected men than in general population (Table 3). In men, the highest SIRs were for Kaposi sarcoma (SIR, 349.10; 95% CI, 196.10-502.20) and anal cancer (SIR, 104.20; 95% CI, 55.56-149.90). The incidence of non-Hodgkin lymphoma (SIR, 15.62; 95% CI, 11.85-19.39), Hodgkin lymphoma (SIR, 16.67; 95% CI, 4.32-29.02), and oropharyngeal cancer (SIR, 2.97; 95% CI, 1.36-4.58) in men infected with HIV was higher than that in the general population. In HIV-infected women, there was an increased incidence of cervical cancer (SIR, 4.98; 95% CI, 1.29-8.66) and non-Hodgkin lymphoma (SIR, 11.78; 95% CI, 2.35-21.21) compared with the general population. The SIR of Hodgkin lymphoma in women was 29.74 (95% CI, 0.00-88.04), but statistical significance was not observed because of the small number of cases. The SIR of thyroid cancer was lower than that of the general population in both men (SIR, 0.63; 95% CI, 0.27-0.99) and women (SIR, 0.48; 95% CI, 0.06-0.90).

**Table 3.** SIRs for Cancer in 467 Patients Infected With HIV 20 to 79 Years of Age in Korea, 2006-2018

Cancer type	Men		Women		
	SIR (95% CI)	*P* value	SIR (95% CI)	*P* value
All cancer	1.68 (1.50-1.87)	<.001	1.26 (0.89-1.64)	.17
AIDS-defining cancer				
Kaposi sarcoma	349.10 (196.10-502.20)	<.001	0	NA
Cervical cancer	NA	NA	4.98 (1.29-8.66)	.04
Non-Hodgkin lymphoma	15.62 (11.85-19.39)	<.001	11.78 (2.35-21.21)	.03
Non–AIDS-defining cancer				
Brain and CNS cancer	0.95 (0.00-2.27)	.94	3.88 (0.00-11.48)	.46
Urinary tract cancer	0.80 (0.28-1.33)	.47	0	NA
Breast cancer	7.76 (0.00-22.98)	.38	0.61 (0.01-1.21)	.20
Colorectal cancer	0.99 (0.61-1.36)	.95	0.63 (0.00-1.51)	.41
Esophagus	0.99 (0.00-2.12)	.99	0	NA
Hodgkin lymphoma	16.67 (4.32-29.02)	.01	29.74 (0.00-88.04)	.33
Liver, bile duct, and pancreas cancer	1.02 (0.67-1.38)	.89	2.43 (0.48-4.38)	.15
Lung cancer	1.39 (0.87-1.90)	.14	1.59 (0.00-3.39)	.52
Malignant immunoproliferative diseases	2.46 (0.30-4.61)	.19	0	NA
Oropharyngeal cancer	2.97 (1.36-4.58)	.02	0	NA
Stomach cancer	0.82 (0.52-1.12)	.25	1.33 (0.03-2.64)	.62
Thyroid cancer	0.63 (0.27-0.99)	.04	0.48 (0.06-0.90)	.02
Prostate cancer	1.63 (0.85-2.41)	.11	NA	NA
Anal cancer	104.20 (55.56-149.90)	<.001	0	NA
Others	1.52 (0.93-2.11)	.08	1.33 (0.16-2.50)	.58

Abbreviations: CNS, central nervous system; NA, not applicable; SIR, standardized incidence rate.

Incident cancer types by year since HIV diagnosis are presented in the Figure. AIDS-defining cancer accounts for approximately 40% of all incident cancers cases within 1 year of HIV diagnosis in both men and women. As the number of years since HIV diagnosis increased, the proportion of AIDS-defining cancer cases decreased, and after 5 years since diagnosis, the proportion of AIDS-defining cancer cases accounted for less than 15% in both men and women.

**Figure.**
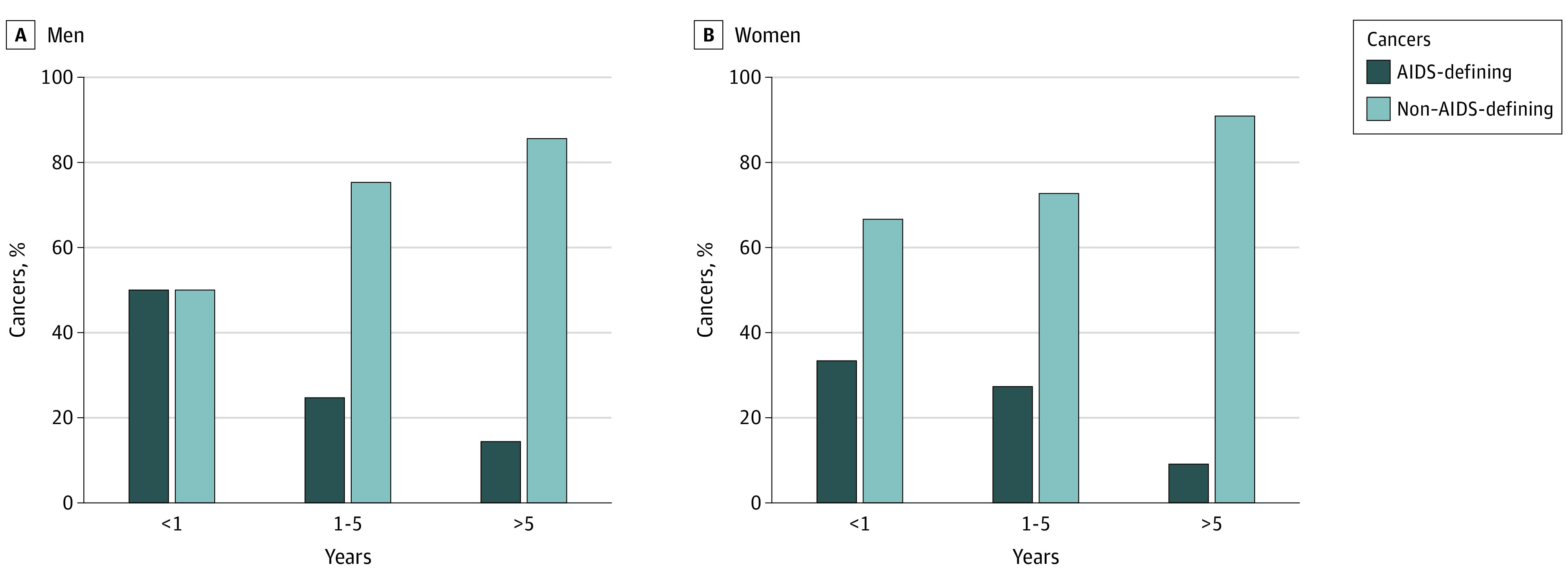
Proportion of AIDS-Defining Cancer Expressed in Years Since HIV Diagnosis

## Discussion

This study identified an increased risk of AIDS-defining cancers and some types of non–AIDS-defining cancers in people with HIV infection in Korea compared with the general population. We observed that both men and women diagnosed with HIV had an elevated risk of AIDS-defining cancers; in men, an increased risk of several non–AIDS-defining cancers, including Hodgkin lymphoma, oropharyngeal cancer, and anal cancer, were noted. However, the incidence of thyroid cancer in men and women with HIV infection was lower than that of the general population.

The increased risk of AIDS-defining cancer was comparable to that reported in previous studies. Despite the introduction of antiretroviral therapy, followed by a decreased incidence of AIDS-defining cancers, the incidence of Kaposi sarcoma, cervical cancer, and non-Hodgkin lymphoma was still higher in people with HIV infection.^8,9,10,11,12,13,14,15^ However, we did not observe a decreasing or increasing pattern of AIDS-defining cancers when the period was divided into 2006-2010, 2011-2015, and 2016-2019. Since 2006, the US Food and Drug Administration approved multiclass combination medicine, and had this study included the period after 2006, the trend might not be observed. A single-center study^16^ in Korea with a study period of 2000 to 2014 also did not find a significant trend in the incidence of AIDS-defining cancers. Long-term studies comparing cancer incidence before and after the introduction of antiretroviral therapy are needed to identify changes in cancer risk according to the improvement of HIV treatment in Korea.

Regarding non–AIDS-defining cancers, the incidence of viral infection–related cancers was significantly higher in Korean people infected with HIV. The incidences of anal and oropharyngeal cancer caused by human papillomavirus (HPV) and Hodgkin lymphoma caused by Epstein-Barr virus were higher in men infected with HIV, as reported in previous studies.^8,9,10,11,14,15,17^ No development of anal or oropharyngeal cancer was observed in women; thus, we did not calculate these SIRs for women. However, unlike findings from previous studies,^8,9,10,11,14,15,17^ our findings suggest that there was no significant increase in liver cancer caused by hepatitis B or C infection and lung cancer caused by smoking or pulmonary diseases in people with HIV in Korea. The prevalence of hepatitis B infection in a Korean HIV/AIDS cohort, including approximately 10% of people infected with HIV in Korea,^18^ was 5.2%, which was similar to that of the general population of men in Korea (4%-5%).^19^ The prevalence of current smoking was comparable between individuals infected with HIV (42.8%)^20^ and Korean men (49.8%).^21^ The similar prevalence of hepatitis B infection and smoking between people infected with HIV and men in the general Korean population might explain the lack of increase in liver or lung cancer incidence among individuals infected with HIV in Korea. Otherwise, most people infected with HIV in Korea are young^4^ and might not be old enough to develop these cancers, showing no increase in liver and lung cancers. Considering the increased number of young people with HIV infection, more extended follow-up studies are needed to identify the risk of age-related cancers.

Despite the higher incidence of endocrine alterations, including thyroid diseases in people with HIV because of the direct effect of HIV viral proteins,^22^ a previous study^23^ suggested that in people with well-treated HIV infection (ie, CD4 cell count of ≤200 cells/mm^3^), the prevalence of symptomatic thyroid dysfunction and thyroid cancer was low. In this study population, the SIR of thyroid cancer in both men and women with HIV infection was lower than that of the general population. According to the 2018 GLOBOCAN estimates, Korea had the highest incidence rates of thyroid cancer worldwide,^24^ and the overdiagnosis attributable to screening was suggested to be the reason for this.^25^ After the issue related to overdiagnosis had been raised, the incidence of thyroid cancer in Korea decreased.^26^ This decrease may reflect a lower frequency of screening tests in people with HIV than in the general population. Despite controversies,^27^ the incidence of cancers for which overdiagnoses have increased, such as prostate cancer and breast cancer, was lower in people with HIV infection than in the general population, suggesting a lower uptake of screening tests among people diagnosed with HIV than in the general population.^28^ In other words, people with HIV infection may have limited medical access, such as primary and secondary prevention. In addition, approximately 40% of incident cancer within 1 year after HIV diagnosis may suggest advanced HIV status at diagnosis, indicating delayed care for HIV diagnosis and treatment. However, the nonsignificantly decreased risk of breast or prostate cancer in people infected with HIV in Korea could be attributed to lower baseline incidence of these cancers in the general population.^26^

One of the notable findings of this study is a distinct cancer incidence pattern between sexes among people infected with HIV in the Korean population. A direct comparison would be difficult because the number of women with HIV was too small, followed by a small number of cancer incidences. However, Kaposi sarcoma, oropharyngeal cancer, and anal cancer are frequent cancers in men infected with HIV but did not develop in women with HIV infection. Most other studies^8,9,11,14,17^ did not stratify sex, but even in a Taiwanese study^15^ based on health claims data, the incidence of Kaposi sarcoma, oropharyngeal cancer, and anal cancer was significantly increased in women with HIV infection compared with the general population. Most of the transmission routes of HIV infection in Korea are sex related, and other causes are rare.^29^ In a study in Taiwan,^30^ the prevalence of HPV in men infected with HIV who have sex with men was 70.8%, and anal samples showed the highest prevalence of high-risk HPV (64.7%); the prevalence of high-risk HPV in the oral samples was 18.5%. A previous systematic review^31^ showed that the prevalence of HPV in men infected with HIV who have sex with men was 86.2% at the anal site and 22.5% at the oral site. No studies have investigated the prevalence of HPV in people with HIV in Korea, but the prevalence of HPV infection was 34.2%, and only 17.5% of adult women had high-risk HPV types, which is lower than the prevalence in men infected with HIV who have sex with men^32^; homosexual and bisexual contacts accounted for 63.5% of the HIV transmission mode in Korea.^33^ Thus, it could be estimated that the HPV prevalence in men with HIV infection would be much higher than that of women, leading to increased cancers caused by HPV infection.

### Limitations

Potential limitations of this study should be considered. First, we stratified the results by sex and standardized age to eliminate the effect of differences in cancer incidence between people infected with HIV and the general population, but other risk factors of cancer, such as smoking, drinking, or sunlight exposure, were not considered because of a lack of information. Second, the NHIS-NHID database is based on claims data, and information on HIV disease markers, such as CD4 cell counts or HIV viral load, were not available. Thus, despite the fact that people with AIDS have a higher risk of AIDS-defining cancers and infection-related cancers,^8^ we did not consider the effect of HIV progression on cancer risk in this study population. However, based on the annual report^3^ on notified HIV/AIDS in Korea, 42.4% of HIV-infected people between 1985 and 2019 reported CD4 cell counts at notification. Of these, 41.7% were in the immunodeficiency group (CD4 cell count <200/μL),^34^ suggesting a high proportion of immunodeficiency or advanced-stage disease in individuals infected with HIV at diagnosis. Third, many people infected with HIV are in the advanced stage of disease when they first visit a clinic; thus, some studies included cancer incidence 5 years before the AIDS period^34^ or 3 years before the first HIV clinic visit.^15^ However, this study considered cancer incidence after 3 months or more at the first HIV clinic visit, as did some previous studies.^8,27^ Most studies regarding cancer risk in people with HIV did not distinguish the risk of incident cancer from the risk of prevalent cancer; however, this study tried to identify cancer risk after HIV diagnosis, despite the advanced stage of disease when diagnosed. This approach might induce an underestimation of cancer risk in individuals infected with HIV. Despite possible underestimation, our results were comparable to those of previous studies.^8,9,11,14,15,17^ Fourth, because of the relatively short time frame and start year of 2006, cancer risks before the introduction of antiretroviral therapy could not be identified. We compared the incidence of several types of cancer together, which might raise the issue of multiple comparison. Despite these limitations, a major strength of this study is its population-based design, covering representatively almost all HIV-infected cases in Korea.

## Conclusions

In this cohort study, cancer risks, especially for AIDS-defining cancer and virus-related cancer, were elevated in people with HIV compared with the general population in Korea. Furthermore, with the increasing number of people newly diagnosed with HIV and the aging of people with HIV, the burden of cancer is expected to increase. Therefore, additional research into prevention, early detection of cancer, and medical accessibility for people living with HIV are essential to reduce cancer morbidity and mortality in this vulnerable population.
