# Error in Affiliations

**DOI:** 10.1001/jamanetworkopen.2022.38976

**Published:** 2022-10-10

In the Original Investigation titled “Cancer Incidence Among Adults With HIV in a Population-Based Cohort in Korea,”^1^ published August 2, 2022, the affiliation for author Won Suk Choi was listed incorrectly; Dr Choi’s correct affiliation is the Division of Infectious Diseases, Department of Internal Medicine, Korea University College of Medicine, Seoul, Republic of Korea. This article has been corrected.^1^
